# Are Colon Polyp Characteristics Really Different Between Men and Women?

**DOI:** 10.1002/jgh3.70161

**Published:** 2025-04-10

**Authors:** Guo‐Jeng Tan, Sanjiv Mahadeva

**Affiliations:** ^1^ Division of Gastroenterology, Department of Medicine, Faculty of Medicine University Malaya Kuala Lumpur Malaysia

**Keywords:** colon polyp, epidemiology, gender

Colorectal cancer affects men differently from women. The sex‐related differences in the incidence of colorectal cancer are well documented. The age‐standardized incidence of colorectal cancer for males in Western Europe in the year 2020 was 20.0 per 100 000; whereas for women, it was 15.1 per 100 000 [1]. In addition to that, adult males suffer more morbidity from colorectal cancer compared with females. In a study among men with colorectal cancer attributable to high body‐mass index, the age‐standardized disability‐adjusted life years rate (ASDR) was 31.09 years per 100 000 people (95% CI 13.36–49.47), compared with women who had an ASDR of only 23.96 years per 100 000 persons (95% CI 10.36–37.75) [2]. This means men lose more years to disability caused by colorectal cancer compared with women. However, it can be argued that there is no significant difference because of the 95% confidence interval overlap. This illustrates the general trend that men are more affected than women. Male patients with CRC also have a poorer survival compared with females. In a cohort study in Australia, the 15‐year survival for males with Stage I disease was reported to be 56.0%, compared with a survival of 68.0% in females. A similar pattern was also seen for Stage II and Stage III disease. Only Stage IV disease had no difference in survival between males and females [3].

Currently, the most widely accepted paradigm for the pathogenesis of colorectal cancer is the adenoma‐carcinoma sequence outlined by Fearon and Vogelstein [4]. This makes the detection of adenomatous polyps vital in the early detection and management of colorectal cancer. Several reports have suggested that, just like CRC, there may be gender differences in the characteristics of colon polyps. A case–control study comparing 794 patients with polyps with 708 colonoscopy‐negative controls found that the male sex was significantly associated with an increase in all polyps [5]. Another study of 5254 subjects identified that the male gender was an independent predictor of adenomas and had a 1.55 (95% CI: 1.02–3.23) odds of developing advanced adenomas [6]. Not only are men more likely to have adenomas, but they are also more likely to have larger adenomas compared with women [7]. There are also gender differences in the location of polyps in the colon, with a study demonstrating that males have an increased 1.88 (95% CI: 1.73–2.04) odds for proximal adenoma and an increased 1.81 (95% CI: 1.67–1.96) odds for distal adenomas [8].

Most of the above studies were conducted in adults undergoing direct colonoscopy without prior stool test screening. Anderson and colleagues' study, as published in JGH Open [9], attempts to explore if the type of polyps and anatomical location of the polyps can be predicted using multi‐target stool DNA (mt‐sDNA) and fecal immunochemical test (FIT). Patients from the New Hampshire Colonoscopy Registry (NHCR) with either positive mt‐sDNA or FIT who then have a colonoscopy were compared with patients who only have direct screening colonoscopies. The baseline demographic and clinical data showed that a positive FIT or mt‐sDNA increases the yield of finding a lesion, regardless of gender, anatomical position (left‐sided or right‐sided lesion), or type of lesion (adenoma or serrated polyp). After statistical adjustments, mt‐sDNA still performed well in all combinations, but FIT fell short in right colonic lesions for males and left colonic advance adenoma for women.

In terms of the demographic characteristics of the men and women in this study, it appears that they were not well matched. The patients with positive mt‐sDNA and FIT tended to be older, were current smokers, and were consuming “blood thinners.” It is not clear whether the “blood thinners” were antiplatelet therapy or anticoagulants. There is evidence that this may not affect FIT [10] but the effects on mt‐sDNA are still unclear. Another issue was that aspirin has been shown to reduce the long‐term risk of colorectal cancer [11] but not the other antiplatelets or anticoagulants, so by lumping everyone together important information may have been missed, even if with adjustment by statistical analysis. Another issue in the cases without FIT or mt‐sDNA were that they had a higher proportion of previous colonoscopies and a family history of CRC. So there is a possibility that patients in this group would have had lesions/polyps removed previously and this would have artificially reduced the yield of advanced lesions, when compared with subjects who had positive stool tests. There were only 1760 patients with positive stool tests compared with 68 645 patients who were screened with colonoscopy only. This created an imbalance that might have rendered some of the results invalid. This was reflected by the narrower confidence intervals for the colonoscopy‐only screening group compared with those with positive stool tests. Another limitation of the study was the division of patients with positive stool tests into smaller categories, which may have led to Type I statistical errors.

In summary, the study by Anderson et al. have shown that positive stool tests correlate more with right‐sided advanced lesions in females but not in males. However, when positive stool tests were used to predict right‐sided advanced adenomas, they were not able to do so in both males and females. Mt‐sDNA appears to predict right‐sided advanced serrated polyps in both males and females, but FIT can only do so for females. The study does show some gender differences in the anatomical position of colonic polyps in patients with a positive stool test. However, the differences are small, and even in combinations that do show a difference, the wide and overlapping 95% confidence intervals seem to preclude any daily practical use when compared to a protocol‐driven colonoscopy done for the correct indication and with good bowel preparation. In closing, we would like to reiterate that the differences between males and females in colorectal cancer are well‐established and well‐recognized. There are also known gender differences concerning polyps; however, we feel that this study was not able to demonstrate how positive stool tests can predict this difference as clearly as the authors would have liked.

## Conflicts of Interest

Prof. Sanjiv Mahadeva is an Editorial Board member of *JGH Open* and a co‐author of this article. To minimize bias, he was excluded from all editorial decision‐making related to the acceptance of this article for publication.
